# Adenovirus-Based Vaccine with Epitopes Incorporated in Novel Fiber Sites to Induce Protective Immunity against *Pseudomonas aeruginosa*

**DOI:** 10.1371/journal.pone.0056996

**Published:** 2013-02-20

**Authors:** Anurag Sharma, Anja Krause, Yaqin Xu, Biin Sung, Wendy Wu, Stefan Worgall

**Affiliations:** 1 Department of Genetic Medicine, Weill Cornell Medical College, New York, New York, United States of America; 2 Department of Pediatrics, Weill Cornell Medical College, New York, New York, United States of America; University of North Dakota, United States of America

## Abstract

Adenovirus (Ad) vector-based vaccines displaying pathogen-derived epitopes on Ad capsid proteins can elicit anti-pathogen immunity. This approach seems to be particularly efficient with epitopes incorporated into the Ad fiber protein. Here, we explore epitope insertion into various sites of the Ad fiber to elicit epitope-specific immunity. Ad vectors expressing the 14-mer *Pseudomonas aeruginosa* immune-dominant outer membrane protein F (OprF) epitope 8 (Epi8) in five distinct sites of the Ad5 fiber, loops CD (AdZ.F(CD)Epi8), DE (AdZ.F(DE)Epi8), FG (AdZ.F(FG)Epi8), HI (AdZ.F(HI)Epi8) and C terminus (AdZ.F(CT)Epi8), or the hexon HVR5 loop (AdZ.HxEpi8) were compared in their capacity to elicit anti-*P. aeruginosa* immunity to AdOprF, an Ad expressing the entire OprF protein. Intramuscular immunization of BALB/c mice with AdZ.F(FG)Epi8 or AdZ.F(HI)Epi8 elicited higher anti-OprF humoral and cellular CD4 and CD8 responses as well as enhanced protection against respiratory infection with *P. aeruginosa* compared to immunization with AdZ.F(CD)Epi8, AdZ.F(DE)Epi8, AdZ.F(CT)Epi8 or AdZ.HxEpi8. Importantly, repeat administration of the fiber- and hexon-modified Ad vectors boosted the OprF-specific humoral immune response in contrast to immunization with AdOprF. Strikingly, following three doses of AdZ.F(FG)Epi8 or AdZ.F(HI)Epi8 anti-OprF immunity surpassed that induced by AdOprF. Furthermore, in the presence of anti-Ad5 immunity, immunization with AdZ.F(FG)Epi8 or AdZ.F(HI)Epi8, but not with AdOprF, induced protective immunity against *P. aeruginosa*. This suggests that incorporation of epitopes into distinct sites of the Ad fiber is a promising vaccine strategy.

## Introduction

*Pseudomonas aeruginosa* is one of the leading nosocomial bacterial pathogens worldwide and can cause serious infections of the respiratory tract. A vaccine against *P. aeruginosa* would be useful as treatment is often challenged by antibiotic resistance of the organism. No efficient and marketable vaccine is yet available [1], [2]. *P. aeruginosa* outer membrane protein F (OprF) is one of the promising vaccine antigens. OprF is surface exposed, antigenically conserved in wild-type strains of *P. aeruginosa* and elicits cross-reactive, opsonizing and protective antibodies in various animal models and humans [1], [3]–[7]. Various immunogenic peptides have been identified in the outer loops of OprF, including the 14-mer peptide Epi8 [8]–[10].

Adenovirus (Ad) vectors are attractive delivery vehicles for genetic vaccines due to their ability to act as immune system adjuvants and to rapidly evoke robust immune responses against the transgene product and viral capsid proteins [11]–[14]. Ad vectors could also serve as a vaccine platform against *P. aeruginosa*. Human Ad serotype 5 (Ad5) or non-human primate Ad serotype C7 (AdC7) expressing OprF induced robust protective immunity against pulmonary infections with *P. aeruginosa* in mice [15], [16].

One of the limitations of Ad as vaccine carrier is that anti-Ad immunity elicited by the initial immunization usually prevents productive infection with subsequent immunizations, critical to achieve boosting of the anti-transgene immunity [13], [17], [18]. One of the prime-boost strategies for Ad-based vaccines is to incorporate vaccine epitopes into the Ad capsid [10], [19]–[22]. Various Ad outer capsid proteins including hexon, fiber knob, penton base and protein IX have been targets for genetic modification [23]. Incorporation of influenza hemagglutinin (HA) epitopes into the fiber HI loop of the Ad5 fiber elicits stronger humoral and cellular immunity compared to incorporation of the same epitope into the more abundant hexon protein [20]. Here we explore different epitope-insertion sites within the Ad fiber protein to enhance the epitope-specific immune response of an Ad-based vaccine. We identify a novel site in the FG loop for epitope insertion to elicit robust epitope-specific immunity that can be boosted and is effective in Ad pre-immune animals.

## Materials and Methods

### Ethics statement

All animal studies were conducted in accordance to the protocols reviewed and approved by the Weill Cornell Institutional Animal Care and Use Committee (Permit Number 0703-594A). All efforts were made to minimize suffering to the animals.

### Ad vectors

**Figure 1:**
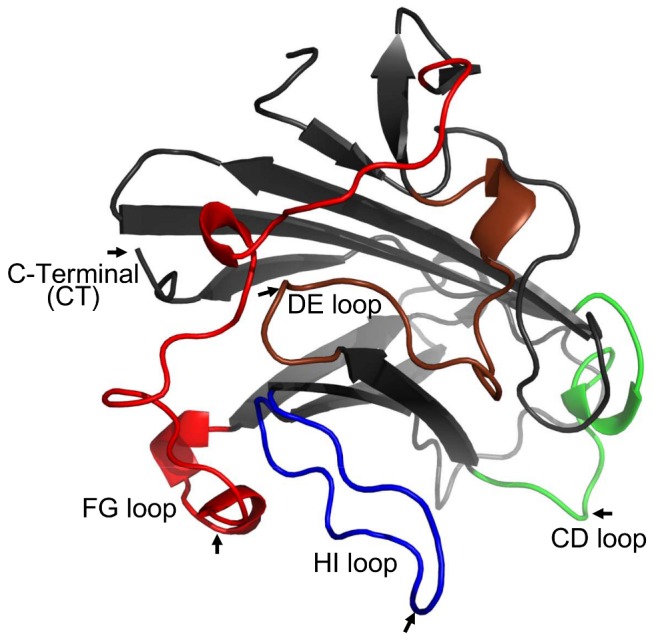
Epitope insertion sites in Ad5 fiber knob. The arrow indicates the location of *P. aeruginosa* OprF 14-mer epitope Epi8 inserted into loops CD (Green; Gly450/Thr451), DE (Brown; Asn464/Gly465), FG (Red; Gly509/Lys510), HI (Blue; Gly543/Asp544 ) and C terminus (CT) of the Ad5 fiber protein (PDB 1KNB).

AdEasy™ adenoviral vector system (Agilent Technologies, Santa Clara, CA) was used to construct the replication-defective recombinant human Ad5 vectors. The vectors expressed either β-galactosidase, referred to as “Z” in the vector (AdZ), or no transgene (AdNull) [24]. The plasmid pAdEasy-1 (Agilent Technologies) was modified to insert gene encoding OprF 14-mer epitope Epi8 (NATAEGRAINRRVE) into loops CD (Gly450/Thr451), DE (Asn464/Gly465), FG (Gly509/Lys510), HI (Gly543/Asp544 ) or C terminus (CT) of the Ad5 fiber gene (**Figure 1**). The resultant plasmids and pAdEasy-1 were recombined with pShuttle-CMV-lacZ (Agilent Technologies) to obtain recombinant plasmids pAdZ.F(CD)Epi8, pAdZ.F(DE)Epi8, pAdZ.F(FG)Epi8, pAdZ.F(HI)Epi8, pAdZ.F(CT)Epi8 and pAdZ that were used for transfection to generate the fiber-modified Ad vectors AdZ.F(CD)Epi8, AdZ.F(DE)Epi8, AdZ.F(FG)Epi8, AdZ.F(HI)Epi8, AdZ.F(CT)Epi8 and AdZ respectively. Fiber-modified Ad vectors were generated using a previously described strategy [25]. Because of potential inhibitory effects of the modified Ad fibers with the cellular Ad receptors, it is difficult to generate fiber-modified vectors in regular human embryonic kidney (HEK) 293 cells. Therefore, a HEK 293-derived cell line that constitutively expresses the Ad5 fiber protein (293F) was developed and used as packaging cell line. The 293F cell line was generated by transfection of a fiber-expressing plasmid (pcDNA3.1/Hyg-Fiber) and then screening single cell clones under hygromycin selection pressure. In addition to the modified fiber protein derived from the viral DNA, Ad vectors generated in 293F cells also carry the wild-type fiber proteins derived from the cell line and can thus effectively infect the unmodified HEK 293 cells. Fiber-modified Ad vectors generated in 293F cells were subsequently propagated in HEK 293 cells to recover the viruses that only carry the modified fiber proteins. AdZ.HxEpi8 has Epi8 inserted into loop 1 of the hypervariable region 5 (HVR5) [10] and AdOprF expresses entire OprF as a transgene [16] as previously described. The vectors were used with equal physical particle concentrations (pu) and were propagated, purified and quantified as described previously [26], [27].

### Mice

Female BALB/c mice were obtained from Taconic Farms. The animals were housed under specific pathogen–free conditions and were used at 6–8 weeks of age. Mice were immunized by injection of 50 µl of the Ad vectors AdZ.F(CD)Epi8, AdZ.F(DE)Epi8, AdZ.F(FG)Epi8, AdZ.F(HI)Epi8, AdZ.F(CT)Epi8, AdZ.HxEpi8, AdOprF, AdZ or AdNull at a dose of 10^10^ pu/animal diluted in PBS to the left thigh muscle using a 0.5 ml insulin syringe (Becton, Dickinson and Company, Franklin Lakes, NJ).

### Western analysis

To evaluate the presence of the Epi8 epitope on the Ad fiber protein, purified Ad vectors (10^10^ virus particles) were denatured (95°C for 5 min) in NuPAGE sample buffer (Invitrogen, Carlsbad, CA) and separated by 4 to 12% gradient sodium dodecyl sulfate-polyacrylamide gel electrophoresis (SDS-PAGE; NuPAGE system; Invitrogen). Following transfer to a polyvinylidene difluoride membrane (Bio-Rad Laboratories, Hercules, CA) equal loading was confirmed by GelCode silver staining (Pierce, Rockford, IL). The membrane was exposed to blocking solution (5% fat-free milk; Bio-Rad Laboratories) in PBS for 1 h and then incubated with either polyclonal anti-OprF serum, obtained from C57Bl6 mice immunized with recombinant OprF protein (1∶500) [16], or anti-Ad fiber antibody (Abcam, Cambridge, MA) (1∶500) for 1 h. Following addition of a peroxidase-conjugated goat anti-mouse antibody (Sigma-Aldrich, St. Louis, MO) (1∶10,000) for 1 h, a chemiluminescent peroxidase substrate (ECL reagent; Amersham Biosciences, Piscataway, NJ) was used for detection.

### Infection with capsid-modified Ad vectors *in vitro*

To evaluate if incorporation of Epi8 in the different fiber sites interferes with the coxsackie-adenovirus receptor (CAR)-dependent or -independent infection *in vitro*, infection of A549 cells (high expression of CAR) or dendritic cells (DC; low expression of CAR) was analyzed. A549 cells (CCL185; American Type Culture Collection, Manassas, VA), maintained in complete Dulbecco's modified essential medium (DMEM) supplemented with 10% fetal bovine serum, 100 U of penicillin/ml, 100 mg of streptomycin/ml (all from GIBCO BRL, Gaithersburg, MD), were infected with AdZ.F(CD)Epi8, AdZ.F(DE)Epi8, AdZ.F(FG)Epi8, AdZ.F(HI)Epi8, AdZ.F(CT)Epi8, AdZ.HxEpi8 or AdZ (1,000 pu/cell) in low-serum medium (2% fetal bovine serum) for 2 h, washed, and then maintained in complete medium for 24 h. Bone marrow-derived DC were generated from bone marrow precursors as described previously [28]. In brief, bone marrow cells harvested from BALB/c mice were grown in complete RPMI 1640 medium [10% fetal bovine serum, 100 U of penicillin/ml, 100 µg of streptomycin/ml supplemented with 10 ng/ml recombinant mouse granulocyte-macrophage colony-stimulating factor (GM-CSF) and 2 ng/ml recombinant mouse interleukin-4 (IL-4) (both from R&D Systems, Minneapolis, MN) for 6–7 days. The DC were then washed and suspended in PBS, infected with the Ad vectors (50,000 pu/cell) for 4 h, and washed and maintained in complete medium for 36 h. The infected A549 cells and DCs were harvested and β-galactosidase activity evaluated using the β-gal assay kit (Invitrogen) as per manufacturer's instructions.

### Anti-OprF, anti-Ad and anti-β-galactosidase humoral immune response

Mice were immunized intramuscularly with AdZ.F(CD)Epi8, AdZ.F(DE)Epi8, AdZ.F(FG)Epi8, AdZ.F(HI)Epi8, AdZ.F(CT)Epi8, AdZ.HxEpi8, AdOprF or AdZ at a dose of 10^10^ pu/animal and boosted twice with the same virus after 2 and 5 wk. Serum was collected after 2, 5 and 8 wk of initial immunization. Lung bronchioalveolar lavage fluid was collected by intratracheal instillation and aspiration of 0.5 ml PBS, pH 7.4., which was centrifuged at 6000 rpm at 4°C for 10 min and the supernatant was stored at −80°C. Anti-OprF, anti-Ad and anti-β-galactosidase IgG were assessed by ELISA using flat bottomed 96-well EIA/RIA plates (Corning, New York, NY) coated with recombinant OprF (0.5 µg/well) [10], Ad5 (10^9^ pu/well) or β-galactosidase (0.1 ug/well) in 0.05 M carbonate buffer, pH 7.4. The plates were blocked with 5% dry milk in PBS for 1 h at 25°C and two-fold serial serum dilutions were added to each well and incubated for 1 h at 25°C. Following three washes with PBS containing 0.05% Tween (PBS-Tween) a peroxidase-conjugated sheep anti-mouse IgG (Sigma-Aldrich), diluted 1∶10,000 in PBS containing 1% dry milk, was added and incubated for 1 h at 25°C. Absorbance at 415 nm was measured with a microplate reader (Bio-Rad Laboratories) and the antibody titers were calculated with a log (OD)–log (dilution) interpolation model and a cutoff value equal to 2-fold the absorbance of the background.

To assess surface exposure of Epi8 on the capsid-modified Ad vectors, ELISA plates were coated with intact or disrupted AdZ.F(CD)Epi8, AdZ.F(DE)Epi8, AdZ.F(FG)Epi8, AdZ.F(HI)Epi8, AdZ.F(CT)Epi8, AdZ.HxEpi8 or AdZ (10^10^ pu/well). Disruption of Ad5 vector was carried out in 0.5% sodium dodecyl sulfate (56°C, 45 seconds) [29]. The plates were blocked with 5% dry milk in PBS for 1 h at 25°C and two-fold serial dilutions of anti-OprF or anti-Ad5 were added to each well and incubated for 2 h at 25°C. The plates were further processed and evaluated as described above.

### OprF-specific cellular immune response

To evaluate OprF-specific cellular immune responses induced by the capsid-modified Ad vectors, BALB/c mice were immunized intramuscularly with 10^10^ pu of AdZ.F(CD)Epi8, AdZ.F(DE)Epi8, AdZ.F(FG)Epi8, AdZ.F(HI)Epi8, AdZ.F(CT)Epi8, AdZ.HxEpi8, AdOprF or AdZ and boosted after 2 and 5 wk with the same vector. The frequency of OprF-specific CD4 and CD8 T lymphocytes was determined with a IL-4- and/or interferon-γ (IFN-γ)-specific enzyme-linked immunospot (ELISPOT) assay (R&D Systems) 7 days following the last immunization. Splenic CD4 or CD8 T cells were purified by positive selection with CD4 (L3T4) or CD8 (Ly-2) MACS microbeads (Miltenyi Biotec, Auburn, CA). The purity of the T cells was more than 95%. To serve as antigen-presenting cells, splenic DCs were purified from syngeneic naive animals by positive selection with CD11c MACS beads (Miltenyi Biotec) and two consecutive purifications over MACS LS columns (Miltenyi Biotec). The purity of the DC was more than 90%. DC (5×10^6^/ml) were incubated for 2 h with purified recombinant OprF protein (100 µg/ml) in RPMI medium supplemented with 2% fetal bovine serum (HyClone, Logan, UT), 10 mM HEPES (pH 7.5; BioSource International, Camarillo, CA), and 10^5^ µM β-mercaptoethanol (Sigma-Aldrich). CD4 or CD8 T cells (2×10^5^) were incubated with splenic DC with or without recombinant OprF protein at a ratio of 4∶1 in IL-4 and/or IFN-γ plates (R&D Systems) for 48 h. Following washing, biotinylated anti-IFN-γ or anti-IL-4 (both from R&D Systems) antibodies were added and incubated overnight at 4°C. For final spot detection a streptavidin-alkaline phosphatase conjugate followed by 3-amino-9-ethylcarbazole substrate (both R&D Systems) was added. The spots were counted by computer-assisted ELISPOT image analysis (Zellnet Consulting, New York, NY).

### Protection against pulmonary challenge with *P. aeruginosa*

The *P. aeruginosa* strain PAO1 was used to assess protective immunity. PAO1-containing agar beads were prepared based on the method of Starke et al. [30] and were used as described previously [31], [32]. Briefly, a log-phase culture of PAO1 suspended in warm tryptic soy agar (52°C) was added to mineral oil with vigorous stirring and the mixture was cooled on ice. The PAO1 -impregnated beads were washed extensively with PBS, and the density of viable bacteria enmeshed in agar beads was determined by plating of serial dilutions of homogenized beads. To evaluate if immunization with Epi8 capsid-modified Ad vectors resulted in protective immunity against a pulmonary challenge with *P. aeruginosa*, BALB/c mice were immunized intramuscularly with AdZ.F(CD)Epi8, AdZ.F(DE)Epi8, AdZ.F(FG)Epi8, AdZ.F(HI)Epi8, AdZ.F(CT)Epi8, AdZ.HxEpi8, AdOprF or AdZ (all 10^10^ pu/mouse) followed by boost immunizations after 2 and 5 wk. Eight weeks following initial immunization, the mice were challenged intranasally with PAO1 (4×10^6^ cfu in 50 ul) encapsulated in agar beads. Mice were sacrificed 24 h post challenge and lung homogenates were plated on McConkey agar plates. The numbers of colonies were quantified after 48 h.

### Statistical Analysis

Data are presented as mean ± standard error of the mean (SEM). Statistical analyses were performed using Two-Way ANOVA and statistical significance was determined at p<0.05.

## Results

### Generation and characterization of fiber-modified Ad vectors

**Figure 2:**
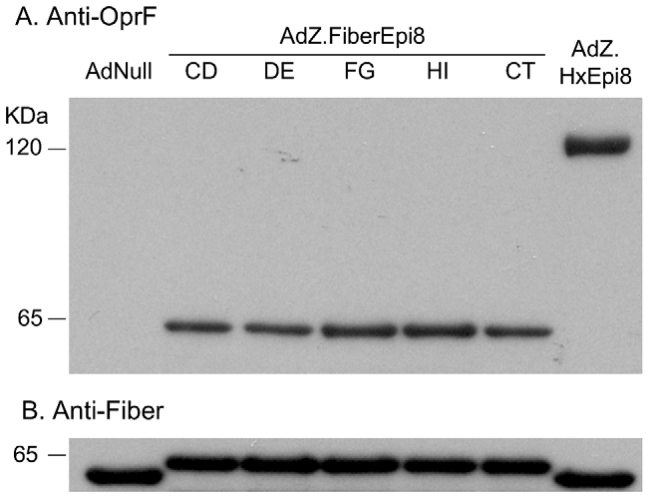
*P. aeruginosa* Epi8 epitope in various Ad vector fiber sites. The fiber-modified Ad vectors AdZ.F(CD)Epi8, AdZ.F(DE)Epi8, AdZ.F(FG)Epi8, AdZ.F(HI)Epi8 and AdZ.F(CT)Epi8, the hexon-modified AdZ.HxEpi8 or AdNull as control (all 10^10^ particles) were separated on 4–12% polyacrylamide gradient SDS-PAGE, transferred to a polyvinylidine difluoride membrane and evaluated by Western blot analysis using **A.** anti-OprF serum; or **B.** anti-Ad fiber antibody.

The Ad vectors with Epi8 incorporated in the fiber loops CD (AdZ.F(CD)Epi8), DE (AdZ.F(DE)Epi8), FG (AdZ.F(FG)Epi8), HI (AdZ.F(HI)Epi8) or C terminal (AdZ.F(CT)Epi8) were generated in the HEK 293-derived cell line that constitutively expresses the Ad5 fiber protein (293F) and subsequently propagated in regular HEK 293 cells as described in material and methods section. The presence of Epi8 epitope in the intact fiber protein on each of the purified vectors was assessed by Western analysis with serum from mice that had been immunized with OprF (**Figure 2A**). Epi8 was detected in the fiber-modified Ad vectors at around 65 kDa, the size of the Ad5 fiber, and in AdZ.HxEpi8 at 120 kDa, the size of Ad5 hexon (**Figure 2A**). No signal was detected with the control AdNull. Detection of the fiber with an anti-fiber antibody showed a slightly increased size of the protein in the five fiber-modified vectors compared to AdNull and Ad.HxEpi8 consistent with the presence of Epi8 (**Figure 2B**).

### Infectivity of Epi8 capsid-modified Ad vectors

**Figure 3:**
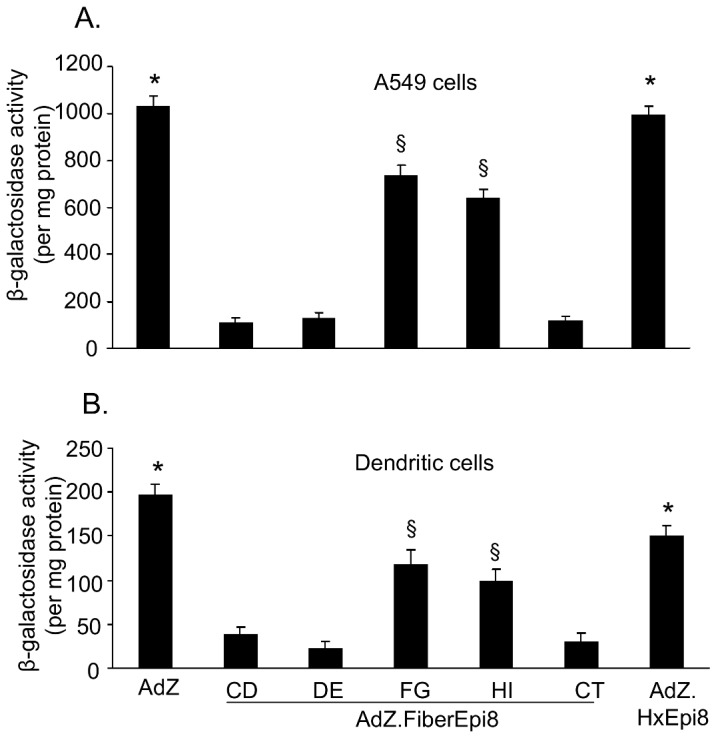
Infectivity of fiber-modified Ad vectors *in vitro*. A549 cells (A) or murine bone-marrow-derived DC (B) were infected with the fiber-modified Ad vectors AdZ.F(CD)Epi8, AdZ.F(DE)Epi8, AdZ.F(FG)Epi8, AdZ.F(HI)Epi8 and AdZ.F(CT)Epi8, the hexon-modified AdZ.HxEpi8 or AdZ as control at 10^3^ pu/cell (A549 cells) or 5×10^4^ pu/cell (DC). β-galactosidase activity was evaluated after 36 h by spectrophotometeric assay and normalized to uninfected cells. Results represent mean ± SEM of three independent experiments. * denotes p<0.05, AdZ or AdZ.HxEpi8 compared to all others. § denotes p<0.05, AdZ.F(FG)Epi8 or AdZ.F(HI)Epi8 compared to AdZ.F(CD)Epi8, AdZ.F(DE)Epi8 or AdZ.F(CT)Epi8.

To evaluate if incorporation of Epi8 into the different fiber sites affected CAR-dependent and CAR-independent infectivity, β-galactosidase (Z) transgene expression was assessed following infection of A549 cells or bone marrow-derived DC. In A549 cells, highest transgene expression was seen with AdZ and AdZ.HxEpi8 (p<0.05, both compared to all other vectors; **Figure 3A**). Of the fiber-modified vectors, AdZ.F(FG)Epi8 and AdZ.F(HI)Epi8 showed the highest expression levels (p<0.05 compared to AdZ.F(CD)Epi8, AdZ.F(DE)Epi8 and AdZ.F(CT)Epi8). Infectivity of DC showed a similar pattern AdZ>Ad.Hx.Epi8>AdZ.F(FG)Epi8 and (AdZ.F(HI)Epi8>AdZ.F(CD)Epi8, AdZ.F(DE)Epi8 and AdZ.F(CT)Epi8 (p<0.05 ) (**Figure 3B**). Overall, these data suggest that incorporation of Epi8 into the fiber protein affects *in vitro* infectivity. Among the five fiber sites, insertion into the FG or HI loop has the least effect on Ad vector infectivity, whereas insertion into the loops CD, DE or CT strongly diminishes Ad infectivity.

### Surface presentation of Epi8 epitope on capsid-modified Ad vectors

**Figure 4:**
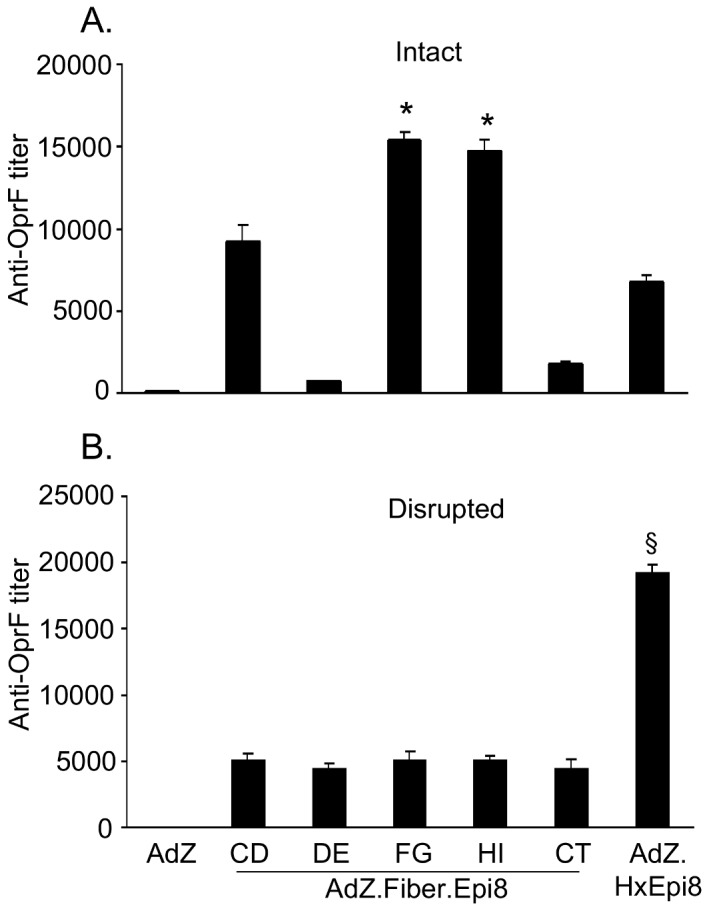
Surface presentation of Epi8 epitope on capsid-modified Ad vectors. ELISA plates were coated with **A.** intact or **B.** disrupted AdZ.F(CD)Epi8, AdZ.F(DE)Epi8, AdZ.F(FG)Epi8, AdZ.F(HI)Epi8, AdZ.F(CT)Epi8, AdZ.HxEpi8 or AdZ (10^10^ pu/well) and probed with anti-OprF serum. Data are shown as the mean ± SEM of 3 wells/vector.* denotes p<0.05, AdZ.F(FG)Epi8 or AdZ.F(HI)Epi8 compared to all others. § denotes p<0.05, AdZ.HxEpi8 compared to all others.

Epitopes inserted in capsid protein could be hidden by protein folding and so affect immune recognition. To investigate surface exposure and accessibility of the Epi8 epitope on the capsid-modified Ad vectors to antibody binding, intact or disrupted Ad vectors were probed with anti-OprF antibody in an ELISA plate. Intact AdZ.F(FG)Epi8 or AdZ.F(HI)Epi8 interacted strongly with anti-OprF compared to intact AdZ.F(CD)Epi8, AdZ.F(DE)Epi8, AdZ.F(CT)Epi8 or AdZ.HxEpi8 (p<0.05; **Figure 4A**). Disrupted fiber-modified Ad vectors interacted at comparable levels with anti-OprF serum (p>0.05; **Figure 4B**). Compared to the disrupted fiber-modified vectors the interaction of disrupted AdZ.HxEpi8 with the OprF serum was stronger (p<0.05), likely secondary to the higher number of hexon units compared to fiber units in an Ad particle (720 *vs* 36, respectively). An anti-Ad antibody was used to assess if even amounts of disrupted and intact vectors were plated (not shown). Overall, these results indicate that Epi8 inserted into the FG or HI is favorably exposed compared to insertion into the CD, DE loops, CT or the loop 1 of HVR5 in hexon.

### Systemic humoral response to Epi8 capsid-modified Ad vectors

**Figure 5:**
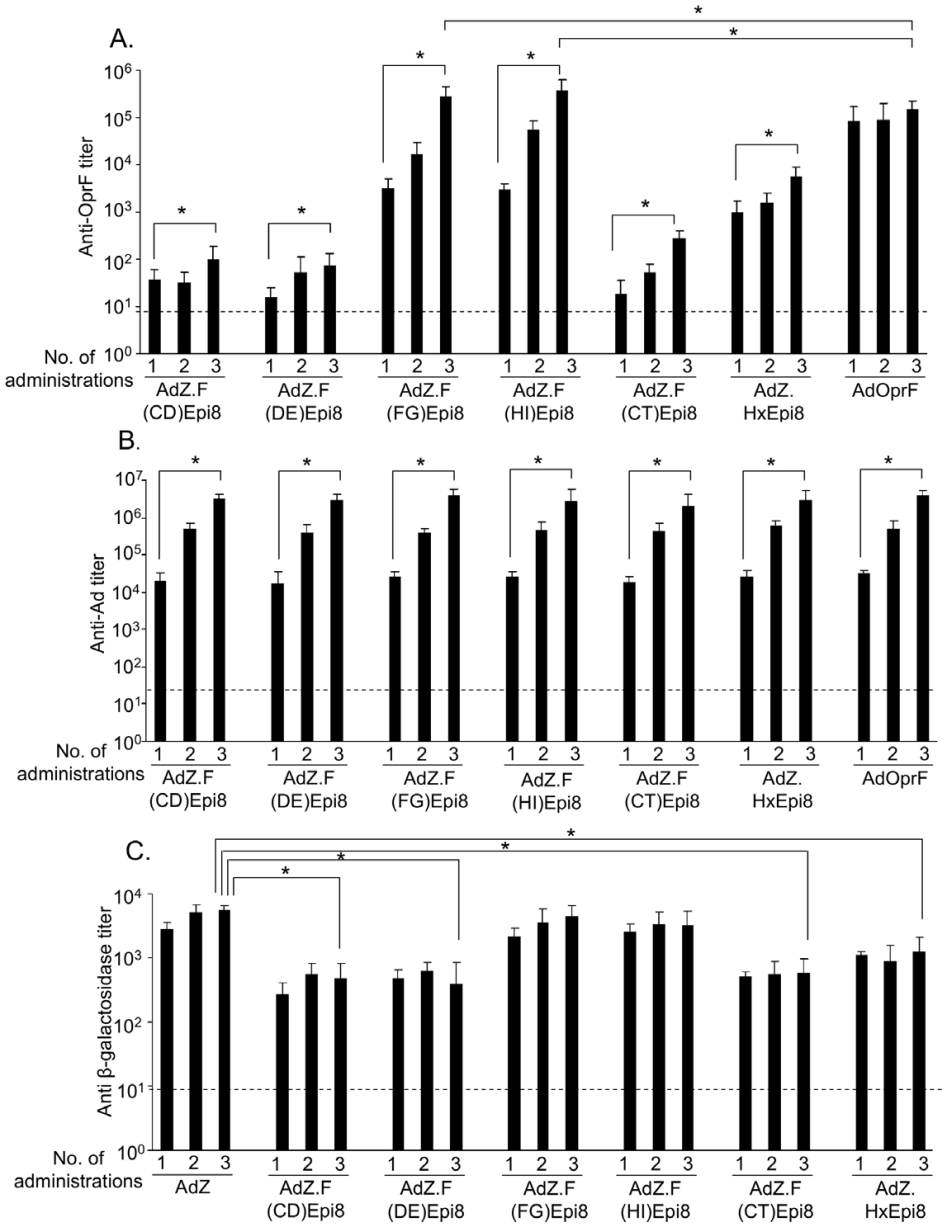
Immunization with Epi8 fiber-modified Ad vectors induces anti-*P. aeruginosa* systemic humoral immunity. BALB/c mice were immunized via the intramuscular route with the fiber-modified Ad vectors AdZ.F(CD)Epi8, AdZ.F(DE)Epi8, AdZ.F(FG)Epi8, AdZ.F(HI)Epi8 and AdZ.F(CT)Epi8, the hexon-modified AdZ.HxEpi8 or AdOprF (all 10^10^ pu/mouse). Mice were boosted with the same vectors after 2 and 5 wk, respectively, and anti-OprF, anti-Ad and anti- β-galactosidase antibodies in serum were analyzed at 2, 5 and 8 wks by ELISA. **A.** Anti-OprF IgG. **B.** Anti-Ad IgG. **C.** Anti-β-galactosidase IgG. Data are shown as the mean ± SEM of 5 mice/group. Limit of detection is indicated by the dashed line. * denotes p<0.05.

To assess the anti-OprF humoral immune response induced by immunization with the Epi8 capsid-modified Ad vectors mice were immunized intramuscularly with either AdZ.F(CD)Epi8, AdZ.F(DE)Epi8, AdZ.F(FG)Epi8, AdZ.F(HI)Epi8, AdZ.F(CT)Epi8, AdZ.HxEpi8, AdOprF or AdZ (10^10^ pu/mouse). At 2 weeks, anti-OprF titers were higher in animals immunized with AdOprF compared to animals immunized with capsid-modified vectors. Immunization with AdZ.F(FG)Epi8 and AdZ.F(HI)Epi8 elicited comparable titers (p>0.05) that were higher compared to immunization with AdZ.F(CD)Epi8, AdZ.F(DE)Epi8, AdZ.F(CT)Epi8 or AdZ.HxEpi8 (p<0.05; **Figure 5A**). Interestingly, repeat administration of the capsid-modified Ad vectors boosted the OprF-specific humoral immune response in contrast to repeat administration of AdOprF (**Figure 5A**). Anti-OprF IgG was higher following three doses of AdZ.F(FG)Epi8 or AdZ.F(HI)Epi8 compared to AdOprF (p<0.05). As expected, anti-Ad IgG titers increased with repeat administration of all vectors (**Figure 5B**). Antibodies against the β-galactosidase transgene were induced by all vectors and were higher with AdZ.F(FG)Epi8 or AdZ.F(HI)Epi8 compared to AdZ.F(CD)Epi8, AdZ.F(DE)Epi8 or AdZ.F(CT)Epi8 (**Figure 5C**). Repeat administration did not result in increase of anti-β-galactosidase titers.

### Lung mucosal humoral immune response to Epi8 capsid-modified Ad vectors

**Figure 6:**
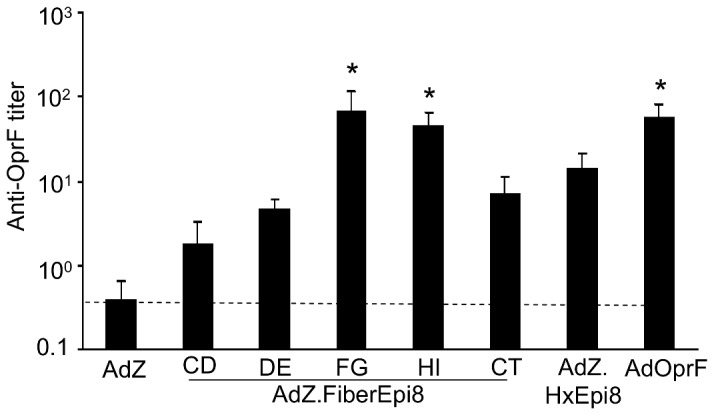
Immunization with Epi8 fiber-modified Ad vectors induces anti-*P. aeruginosa* lung mucosal humoral immunity. BALB/c mice were immunized via the intramuscular route with the fiber-modified Ad vectors AdZ.F(CD)Epi8, AdZ.F(DE)Epi8, AdZ.F(FG)Epi8, AdZ.F(HI)Epi8 and AdZ.F(CT)Epi8, the hexon-modified AdZ.HxEpi8, AdOprF or AdZ (all 10^10^ pu/mouse) and boosted with the same vectors at 2 and 5 wk. Anti-OprF IgG in bronchioalveolar lavage fluid was analyzed by ELISA after 8 wk. Data are shown as the mean ± SEM of 5 mice/group. Limit of detection is indicated by the dashed line. * denotes p<0.05, AdZ.F(FG)Epi8, AdZ.F(HI)Epi8 or AdOprF compared to all others.

To assess the lung mucosal anti-OprF humoral immunity, anti-OprF titers were evaluated in bronchioalveolar lavage fluid. Anti-OprF IgG was detected in all mice immunized with the Epi8 capsid-modified or OprF expressing vectors but not in mice immunized with AdZ (**Figure 6**). Immunization with AdZ.F(FG)Epi8, AdZ.F(HI)Epi8 and AdOprF elicited comparable titers that were higher compared to AdZ.F(CD)Epi8, AdZ.F(DE)Epi8, AdZ.F(CT)Epi8 or AdZ.HxEpi8 (p<0.05).

### Cellular immune response to Epi8 capsid-modified Ad vectors

**Figure 7:**
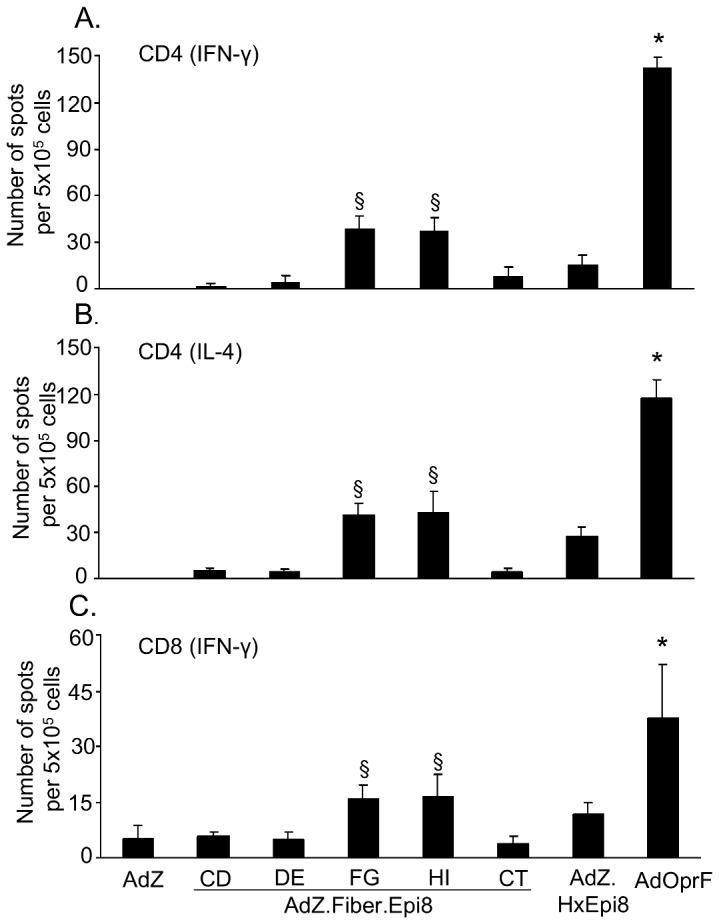
Immunization with Epi8 fiber-modified Ad vectors induces anti-*P. aeruginosa* cellular immunity. BALB/c mice were immunized via the intramuscular route with the fiber-modified Ad vectors AdZ.F(CD)Epi8, AdZ.F(DE)Epi8, AdZ.F(FG)Epi8, AdZ.F(HI)Epi8 and AdZ.F(CT)Epi8, the hexon-modified AdZ.HxEpi8, AdOprF or AdZ (all 10^10^ pu/mouse) and boosted with the same vectors at 2 and 5 wk. Splenic CD4 and CD8 T cells were isolated 7 days following the last administration and incubated *in vitro* with syngeneic DC pulsed with recombinant OprF or DC alone. IL-4 and IFN-γ were determined by ELISPOT assay. **A.** CD4 IFN-γ; **B.** CD4 IL-4; **C.** CD8 IFN-γ. The data represent the mean of pooled cells from five mice per group from three separate experiments ± SEM. * denotes p<0.05, AdOprF compared to all others. § denotes p<0.05, AdZ.F(FG)Epi8 or AdZ.F(HI)Epi8 compared to AdZ, AdZ.F(CD)Epi8, AdZ.F(DE)Epi8, AdZ.F(CT)Epi8 or AdZ.HxEpi8.

To evaluate the cellular immune responses, mice were immunized intramuscularly with AdZ.F(CD)Epi8, AdZ.F(DE)Epi8, AdZ.F(FG)Epi8, AdZ.F(HI)Epi8, AdZ.F(CT)Epi8, AdZ.HxEpi8, AdOprF or AdZ and boosted twice with the same vector. The frequencies of OprF-specific splenic CD4 and CD8 T cells stimulated by syngeneic DC pulsed with recombinant OprF protein were analyzed by ELISPOT. Immunization with AdOprF induced the highest OprF-specific IFN-γ CD4 (**Figure 7A**), IL-4 CD4 (**Figure 7B**) and IFN-γ CD8 T cell (**Figure 7C**) responses (p<0.05). Of the capsid-modified vectors, AdZ.F(FG)Epi8 and AdZ.F(HI)Epi8 induced higher OprF-specific IFN-γ CD4 (**Figure 7A**), IL-4 CD4 (**Figure 7B**) and IFN-γ CD8 T cell (**Figure 7C**) compared to AdZ.F(CD)Epi8, AdZ.F(DE)Epi8, AdZ.F(CT)Epi8 or AdZ.HxEpi8 (p<0.05).

### Protection against pulmonary infection with *P. aeruginosa*

**Figure 8:**
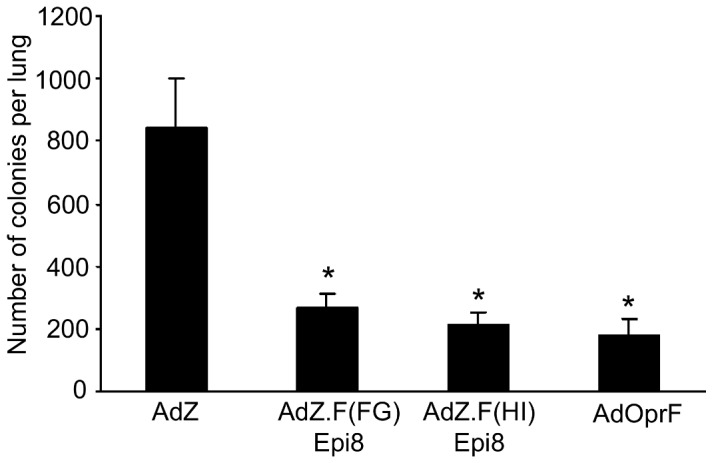
Protective anti-*P. aeruginosa* immunity induced by immunization with Epi8 fiber-modified Ad vectors. BALB/c mice were immunized via the intramuscular route with AdZ.F(FG)Epi8, AdZ.F(HI)Epi8, AdOprF and AdZ at a dose of 10^10^ pu/mouse followed by boost immunizations after 2 and 5 wk. Eight weeks after immunization, mice were challenged with agar-encapsulated *P. aeruginosa* (4×10^6^ cfu). Bacterial counts in lung homogenate were performed after 24 h. Data are shown as means ± SEM of 5 mice/group. * denotes p<0.05, compared to AdZ-immunized mice.

To evaluate if the most immunogenic Epi8 fiber-modified Ad vectors protect against pulmonary infection with *P. aeruginosa*, mice were immunized with AdZ.F(FG)Epi8, AdZ.F(HI)Epi8, AdOprF or AdZ, boosted twice with the same vector and challenged by intranasal administration of agar-encapsulated PAO1 three weeks after the last vector administration. Mice immunized with AdZ.F(FG)Epi8, AdZ.F(HI)Epi8 and AdOprF showed reduction in the *P. aeruginosa* colony count compared to the AdZ group (p<0.05; **Figure 8**). This suggests that the protective immunity generated by AdZ.F(FG)Epi8 or AdZ.F(HI)Epi8 is comparable to that induced by AdOprF.

### Efficacy of Epi8 fiber-modified Ad vectors in the presence of anti-Ad immunity

**Figure 9:**
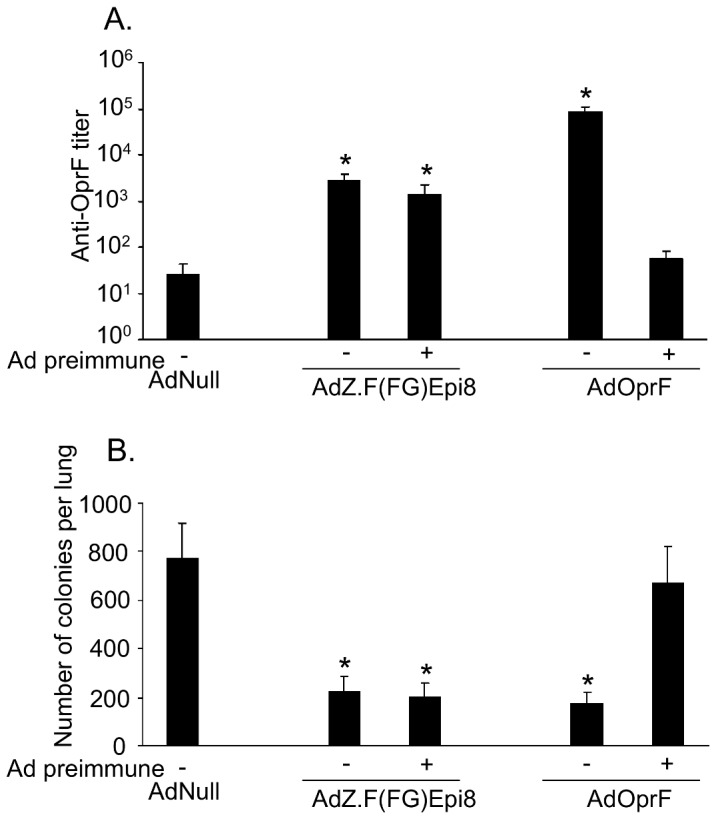
AdZ.F(FG)Epi8 induces robust humoral and protective anti-*P. aeruginosa* in the presence of pre-existing anti-Ad immunity. BALB/c mice were immunized with AdZ.F(FG)Epi8, AdOprF or AdNull (all 10^10^ pu)/mouse in the presence or absence of anti-Ad5 immunity. Mice were inoculated with 10^10^ pu/mouse of AdNull and boosted twice at 2 weeks interval in order to mimic pre-existing immunity **A.** Anti-OprF IgG in serum determined by ELISA at 4 wk. Limit of detection is indicated by dotted line. * denotes p<0.05, compared to AdNull-immunized mice. **B.** Protective immunity against pulmonary challenge with agar-encapsulated *P. aeruginosa* (4×10^6^ cfu) 5 wk after immunization. Bacterial counts in lung homogenate were performed after 24 h. Data are shown as means ± SEM of seven mice per group. * denotes p<0.05, compared to AdNull-immunized mice.

To evaluate the efficacy of fiber-modified Ad vectors in the presence of pre-existing anti-Ad immunity, Ad-immune mice, induced by repeat administration of AdNull, were immunized with AdZ.F(FG)Epi8 or AdOprF. In the presence of pre-existing anti-Ad immunity, AdOprF inoculated mice showed a marked reduction in anti-OprF titers that were close to basal levels (**Figure 9A**). In contrast, immunization with AdZ.F(FG)Epi8 elicited robust levels of anti-OprF IgG irrespective of pre-existing Ad immunity. Likewise, protection against *P. aeruginosa*, was similar when AdZ.F(FG)Epi8 was administered in the presence or absence of anti-Ad immunity (**Figure 9B**). This suggests that, in contrast to AdOprF, AdZ.F(FG)Epi8 can elicit protective anti-*P. aeruginosa* immunity even in the presence of anti-Ad immunity.

## Discussion

A potent and effective vaccine against *P. aeruginosa* has long been sought after, but is so far not available. The present study demonstrates that fiber-modified Ad vectors expressing Epi8 induce anti-*P. aeruginosa* humoral and cellular protective immunity that can be boosted on repeated administration and is effective in presence of anti-Ad5 immunity.

### Incorporation of epitopes into Ad vector capsid to induce epitope-specific immunity

One attractive feature of Ad-based vaccines is the feasibility to modify the Ad capsid to enhance immune responses or change the Ad tropism [23], [33]. Incorporation of Epi8 into loop 1 of HVR5 of the Ad hexon protein has been shown to induce anti-epitope humoral and cellular immunity to protect against infections with *P. aeruginosa* in a murine model [16]. Incorporation of influenza HA or ovalbumin epitopes into various Ad capsid proteins demonstrated that incorporation into the fiber HI loop induces the strongest anti-epitope response [20], [21]. The structure of the Ad5 fiber is known and there are multiple loops and sites that could theoretically be used as insertion sites for peptide sequences without disrupting the overall structure [34]. We developed and compared various Ad vectors that display Epi8 on the CD, DE, FG, HI loops or CT of Ad fiber knob. Immunization with fiber-modified Epi8 Ad vectors induced robust humoral and cellular responses. Following single administration anti-OprF immunity induced by the fiber-modified vectors was less compared to anti-OprF immunity induced by a vector expressing the entire OprF protein as transgene. Among the capsid-modified vectors, the strongest humoral response against the OprF protein was induced by AdZ.F(FG)Epi8 or AdZ.F(HI)Epi8. The AdZ.HxEpi8, AdZ.F(CD)Epi8, AdZ.F(DE)Epi8 and AdZ.F(CT)Epi8 induced only low levels of anti-OprF humoral immunity. The stronger induction of Epi8-specific immune response by fiber-modified Ad vector compared to hexon-modified vector is consistent with our previous observations with HA epitope [20]. The low efficiency of CD, DE fiber loops CT or loop 1 of HVR5 of Ad hexon to generate epitope-specific immune responses can be explained by the interference of the inserted peptide with cellular infectivity, in particular of antigen-presenting cells or the impaired folding and exposure of the epitopes on the capsid. In contrast to our results, higher humoral immunity after single immunization was elicited against an ovalbumin epitope incorporated into the hexon protein compared to insertion of the epitope into the fiber HI loop [21]. It is likely that the nature of the two different epitopes and the location of the epitope within the hexon (insertion within HVR5 in this study *versus* replacement of HVR5 by Lanzi *et al.*) influenced the epitope-specific immune responses. Consistent with our results, upon second administration a stronger humoral response was elicited when ovalbumin epitope was inserted into fiber protein compared to hexon [21].

Protective immunity against extracellular bacteria such as *P. aeruginosa* is mainly dependent on sufficient levels of humoral immunity induced by the vaccine. T cell-mediated immunity has received less attention in the development of a vaccine against *P. aeruginosa* but is a part of the response against natural infection with the organism [35], [36]. Consistent with the humoral response, FG- and HI- modified vectors elicited strong OprF-specific Th1 and Th2 type cellular immunity, which was lower compared to AdOprF. The presence of multiple T-cell epitopes in the full length OprF protein used to pulse DCs, is likely responsible for higher T-cell activation by AdOprF. Importantly, the protective immunity generated by AdZ.F(FG)Epi8 or AdZ.F(HI)Epi8 was comparable to AdOprF. Although both humoral and cellular responses were induced after immunization with AdZ.F(FG)Epi8 or AdZ.F(HI)Epi8 in the present study, their individual contribution to the protection against *P. aeruginosa* challenge is not clear.

The HI loop in the fiber knob has been the usual choice for incorporation of antigenic epitopes or targeting moieties [20], [21], [35], [36]. In the present study we identified the FG loop as a novel location in the fiber knob for peptide insertion. This site is comparable to the HI loop regarding *in vitro* infectivity and its capacity to elicit anti-peptide immunity. It would be interesting to assess infectivity and immunogenicity of a bispecific Ad vector with peptides at both FG and HI loops.

### Boosting of anti-OprF immune response by repeat administration of fiber-modified Ad vectors

Anti-Ad immune responses impair efficacy of Ad vectors of the same serotype, as pre-existing neutralizing antibodies against Ad prevent the cellular uptake of Ad and expression of the transgene in a previously immunized host [37], [38]. To circumvent this issue Ad vaccine vectors derived from rare human or nonhuman Ad serotypes that evade anti-Ad5 immunity have been developed [39]–[41]. However, all of these Ad vectors generate potent anti-vector immunity that diminishes the utility of vector re-administration/boosting. Anti-Ad immunity can be boosted by repeated infection with wild-type Ad or Ad vectors, leading to an increase in anti-Ad humoral responses with subsequent infections [37], [38], [42]. Consequently, immune response against a foreign epitope placed on an Ad capsid protein can be boosted by repeated vector administration. Immunization with the capsid-modified Ad vectors in this study enabled repeated administration of the same vector resulting in boosting of the anti-epitope and not the anti-transgene humoral response. Strikingly, anti-OprF IgG was higher following three doses of FG- or HI-modified vector compared to AdOprF, thus highlighting the utility of fiber-modified Ad vectors for vaccine delivery. One of the mechanisms that explains the boosting of humoral response is the Fcγ receptor-mediated uptake of Ad vector-antibody immune complexes by antigen-presenting cells and subsequent increased stimulation of specific immune cells [43].

The prevalence of pre-existing vector immunity in humans may limit the utility of human Ad serotype vaccines. Notably, in sharp contrast to immunization with AdOprF, AdZ.F(FG)Epi8 induced protective anti-*P. aeruginosa* immunity even in the presence of high levels of pre-existing anti-Ad immunity. This suggests that pre-existing Ad vector immunity can be effectively circumvented by the incorporation of antigenic epitopes into the fiber protein. Taken together, incorporation of antigenic peptides on the FG or HI loop of Ad fiber knob is an efficient strategy to generate protective immunity that can be boosted by repeated administration and is effective in the presence of pre-existing anti-Ad immunity. The display of antigenic epitopes on the Ad fiber should also be valuable in the development of Ad-based vaccines against other pathogens. The robust protective immunity induced by AdZ.F(FG)Epi8 and AdZ.F(HI)Epi8 make both of these sites attractive for the insertion of epitopes as a general vaccine strategy.
